# Fractional Laser Releases Tumor-Associated Antigens in Poorly Immunogenic Tumor and Induces Systemic Immunity

**DOI:** 10.1038/s41598-017-13095-8

**Published:** 2017-10-06

**Authors:** Masayoshi Kawakubo, Trevor J. Cunningham, Shadmehr Demehri, Dieter Manstein

**Affiliations:** 1 Cutaneous Biology Research Center, Department of Dermatology, Massachusetts General Hospital Research Institute, Charlestown, MA 02129 USA; 2 Harvard Medical School, Boston, MA USA

## Abstract

Currently ablative fractional photothermolysis (aFP) with CO_2_ laser is used for a wide variety of dermatological indications. This study presents and discusses the utility of aFP for treating oncological indications. We used a fractional CO_2_ laser and anti-PD-1 inhibitor to treat a tumor established unilaterally by the CT26 wild type (CT26WT) colon carcinoma cell line. Inoculated tumors grew significantly slower in aFP-treated groups (aFP and aFP + anti-PD-1 groups) and complete remission was observed in the aFP-treated groups. Flow cytometric analysis showed aFP treatment elicited an increase of CD3+, CD4+, CD8+ vand epitope specific CD8+ T cells. Moreover, the ratio of CD8+ T cells to Treg increased in the aFP-treated groups. Additionally, we established a bilateral CT26WT-inoculated mouse model, treating tumors on one-side and observing both tumors. Interestingly, tumors grew significantly slower in the aFP + anti-PD-1 groups and complete remission was observed for tumors on both aFP-treated and untreated sides. This study has demonstrated a potential role of aFP treatments in oncology.

## Introduction

Fractional photothermolysis (FP) can be characterized as laser-assisted treatment generating a pattern of microscopic treatment zones (MTZs) in biological tissue^1^. There are two FP modes, the non-ablative mode (nFP) and ablative (aFP) mode. nFP generates MTZs as small zones of thermally damaged tissue, whereas aFP additionally produces a central “hole” of physically-removed (ablated) tissue, surrounded by a small cuff of thermally-damaged tissue in MTZs^2,3^. In general, the width or diameter of the MTZs measures less than 0.5 mm. FP techniques typically expose only a small fraction of the tissue (often an areal fraction of approximately 5–27%), leaving the majority of tissue spared or unexposed^4^. A large range of dermatological indications, such as treatment of photodamaged skin, dyschromia, rhytides, and different kind of scars including acne, surgical and burn scars currently make use of FP^1,5–8^. However, FP is not used for the treatment of tumors and no studies to date have investigated production of systemic effects using FP methods.

Mroz *et al*. reported laser irradiation of photodynamic therapy (PDT) to treat mice subcutaneously inoculated with CT26.CL25 colon carcinoma cells inducing local remission and antigen-specific immune response systemically, promoting regression of a remote and untreated tumor^9^. However CT26.CL25 cells are artificially transduced with the lacZ gene to stably express a tumor antigen (beta-gal). One limitation of the study is that the tumor model used is not a clinically relevant tumor, and instead represents an artificially-induced cancerous state in the subject mice. The phenomenon promoting regression of a remote and untreated tumor was not observed when they employed CT26 wild type (CT26WT) undifferentiated colon carcinoma cells, which are beta-gal negative parental carcinoma of CT26.CL25 cells. This is surprising because many reports showed PDT could induce the immune response against locally inoculated CT26WT colon carcinoma cells^10–12^. These carcinoma cells are a clone of the N-nitoroso-N-methylurethan (NMU)-induced grade IV carcinoma, which is rapidly-growing and readily-metastasizing^13^. Therefore we decided to challenge and treat these poorly immunogenic CT26WT colon carcinoma cells with aFP and anti-PD-1 inhibitor, which boosts the function of CD8+ T cells by blocking the PD-1/ PD-L1 pathway^14–16^, to confirm if aFP can be used for induction of anti-tumor immunity and promote regression of a remote and untreated tumor in the clinical relevant situation, which is in marked contrast to the current applications of FP.

## Results

### aFP is significantly effective against local primary tumors

**Figure 1:**
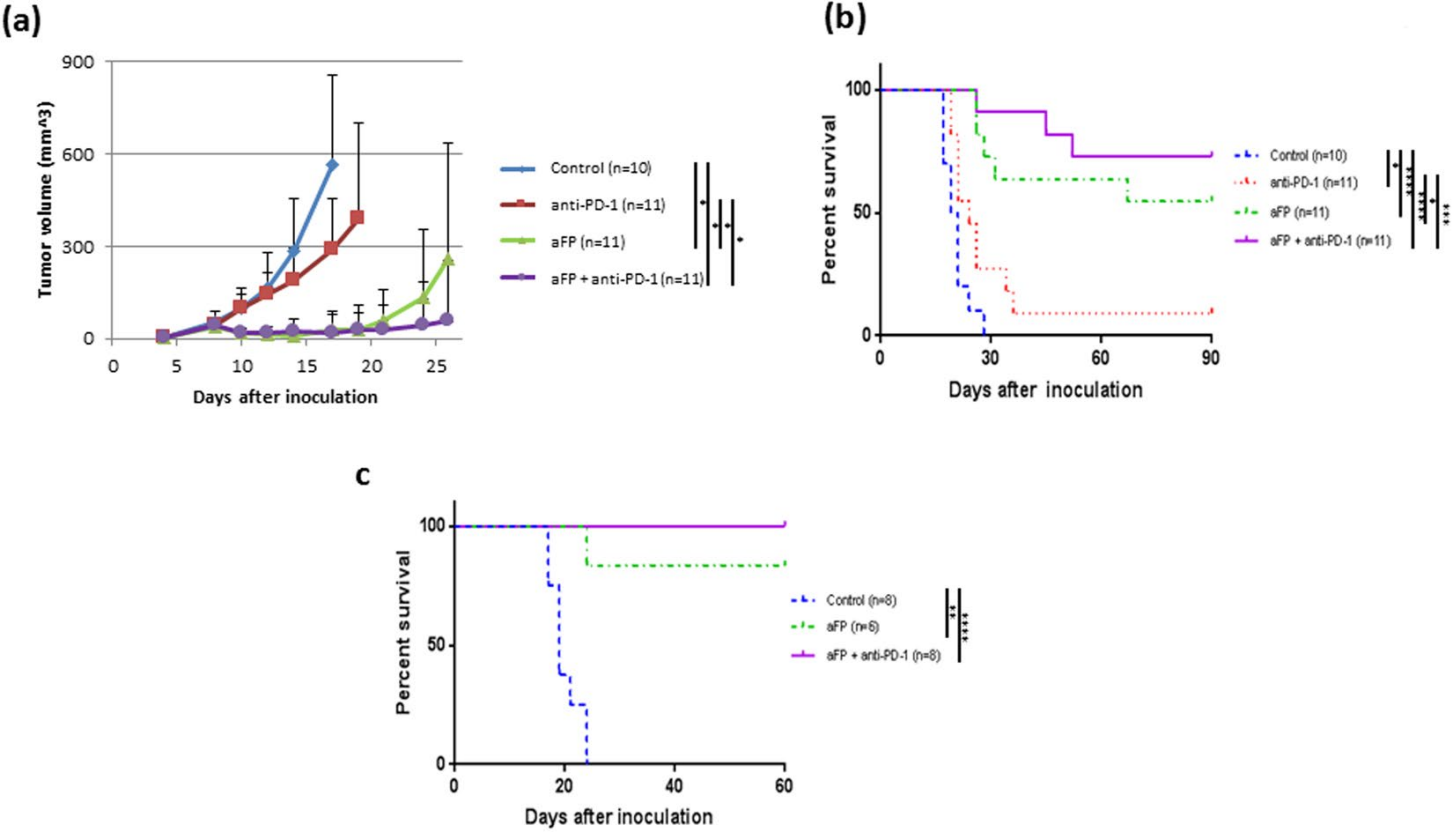
Tumor Volume and Survival Curves after Treatment and Rechallenge Test in the One Tumor Mouse Model. Mice were inoculated unilaterally at the left leg with 3.5 × 10^5^ CT26WT subcutaneously into the depilated thigh. aFP laser irradiation was performed 8 days after tumor inoculation, and then the growth of the tumors was observed. Anti-PD-1 blocking antibodies were administered intraperitoneally at a dose of 200 µg per mouse on days 8, 10, 12, 14, and 16 in the one tumor mouse model after tumor cell inoculation. (**a**) Tumor volume curves of mice in the control, anti-PD-1, aFP and aFP + anti-PD-1 groups after tumor inoculation. (**b**) Kaplan-Meier survival curves of mice receiving tumor inoculation. The significance values for the difference between the survival curves are: Control vs. anti-PD1: *P* < 0.05, Control vs. aFP: *P* < 0.0001, control vs. aFP + anti-PD1: *P* < 0.0001, anti-PD-1 vs. aFP: *P* < 0.05, anti-PD1 vs. aFP + anti-PD1: *P* < 0.0002. (**c**) Kaplan-Meier survival curves of mice receiving the rechallenge test with CT26WT cells. These mice were each inoculated with 3.5 × 10^5^ CT26WT cells subcutaneously in the contralateral (right) thigh against previous inoculation. The significance values for the difference between the survival curves are: control mice vs. survival mice (p < 0.005), control vs. aFP + anti-PD1: *P* < 0.0001. *The bars represent SD. *P* < 0.05, **P < 0.005, ***P < 0.0005, ****P < 0.0001.

aFP laser irradiation was performed 8 days after tumor inoculation. The aFP significantly led to a reduction in tumor volume and growth rate of CT26WT tumors after the treatment (Fig. 1a). Tumors shrank in 6 mice of 11 mice in the aFP group and 8 mice of 11 mice in the aFP + anti-PD-1 group, but such shrinkage occurred in only 1 mouse (9%) from the anti-PD-1 group or does not occur in the control group (Fig. 1b). The significance value for the difference between the survival curves are: control vs. anti-PD-1 (*p* < 0.05), control vs. aFP (*p* < 0.0001), control vs. aFP + anti-PD-1 (*p* < 0.0001), anti-PD-1 vs. aFP (*p* < 0.005) and anti-PD-1 vs. aFP + anti-PD-1 (*p* < 0.0005).

### aFP induces development of long term anti-tumor immunity

To assess the presence of any induced long term anti-tumor immunity, we performed a rechallenge experiment following the treatment. Six mice in the aFP group and eight mice in the aFP + anti-PD-1 group which survived for more than 90 days after the treatment, were subsequently inoculated subcutaneously with CT26WT cells in the contralateral (right) thigh. Eight age-matched naive mice were inoculated with the same number of CT26WT cells, respectively, in the right thigh as a control. The tumor on the naive mice in the control groups and one of the six mice in the aFP group progressed over time. Tumors on the rest of the survival mice in the aFP group and all survival mice in the aFP + anti- PD-1 group did not appear to progress (Fig. 1c). They remained tumor–free for at least another 60 days following the inoculation (Fig. 1c). The significance values for the differences between the survival curves are: control vs. aFP (*p* < 0.001), control vs. aFP + anti-PD-1 (*p* < 0.0001).

### aFP increases CD3+, CD4+ and CD8+ T cell numbers and develop epitope specific CD8+ lymphocytes in aFP-treated tumor

**Figure 2:**
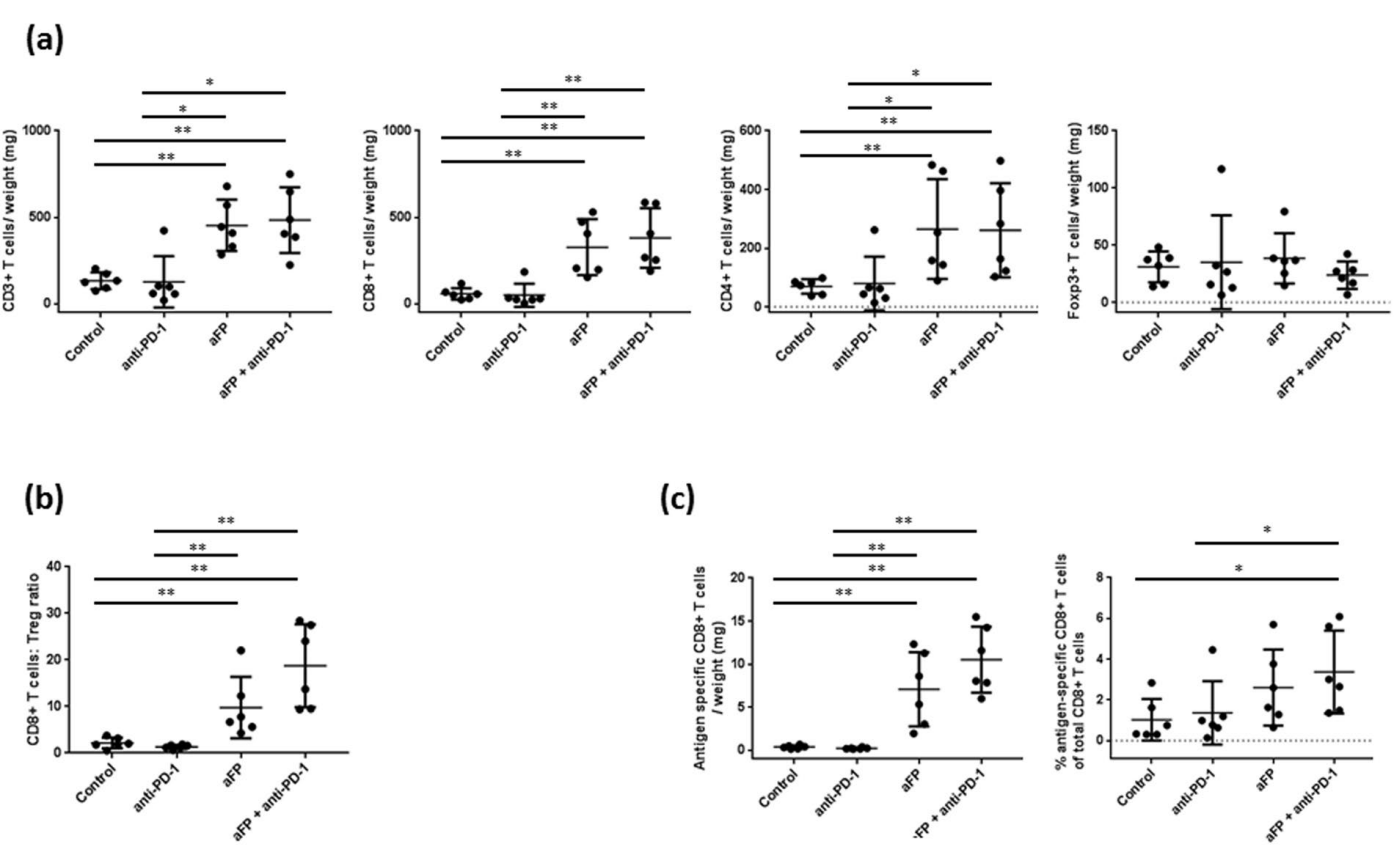
Flow Cytometric Analysis for Tumor Infiltrating Lymphocytes 5 Days After aFP Treatment in the One Tumor Mouse Model. Flow cytometry analysis was performed to confirm whether CD3+ and CD8+ lymphocyte, and regulatory T cell (Treg) numbers were affected by aFP. (**a**) Proportion of CD3+, CD8+ and CD4+ T cells and Treg normalized to tumor weight. (**b**) Ratio of CD8+ T cells to Tregs (CD4+ Foxp3+). (**c**) Proportion of antigen specific CD8+ T cells normalized to tumor weight and the percentage of antigen specific CD8+ T cells normalized to total number of CD8+ T cells. The bars represent SD. * *P* < 0.05, **P < 0.005.

To investigate if the adaptive immune system was affected by aFP, we measured the number of CD3roup and all survival mice in, CD4roup and all survival mice in, and CD8+ T cells and Foxp3+ Tregs inside the tumor using flow cytometry 5 days after aFP treatment. We chose to measure on 5 days after aFP treatment because by this day the aFP-treated tumor in the aFP and aFP + anti-PD-1 groups showed significantly shrinkage. We found that CD3+, CD4+, CD8+ T cells increased per tumor weight in the aFP-treated groups (CD3/weight: control vs. aFP and control vs. aFP + anti-PD-1 (*p* < 0.005), anti-PD-1 vs. aFP and anti-PD-1 vs. aFP + anti-PD-1 (*p* < 0.05); CD4/weight: control vs. aFP, control vs. aFP + anti-PD-1, anti-PD-1 vs. aFP and anti-PD-1 vs. aFP + anti-PD-1 (*p* < 0.005); CD8/weight: control vs. aFP and control vs. aFP + anti-PD-1 (*p* < 0.005), anti-PD-1 vs. aFP and anti-PD-1 vs. aFP + anti-PD-1 (*p* < 0.05; Fig. 2a)). However, there is no significant difference regarding Treg in the all groups (Fig. 2a). Moreover, the ratio of CD8+ T cells compared to Treg also increased in the aFP-treated groups (control vs. aFP, control vs. aFP + anti-PD-1, anti-PD-1 vs. aFP and anti-PD-1 vs. aFP + anti-PD-1 (*p* < 0.005); Fig. 2b). Additionally, to investigate TAA specific CD8+ T cell numbers, we measured AH1 epitope specific CD8+ lymphocytes. We found that aFP led to a significant increase AH1 specific CD8+ T cells per tumor weight in the aFP-treated groups (control vs. aFP, control vs. aFP + anti-PD-1, anti-PD-1 vs. aFP and anti-PD-1 vs. aFP + anti-PD-1 (*p* < 0.005); Fig. 2c). However, the percentage of AH1 specific CD8+ T cells compared with total CD8+ T cells increased in only aFP + anti-PD-1 group (control vs. aFP + anti-PD-1 and anti-PD-1 vs. aFP + anti-PD-1 (*p* < 0.05); Fig. 2c).

### Adaptive immunity is necessary to eradicate cancer cells after aFP

**Figure 3:**
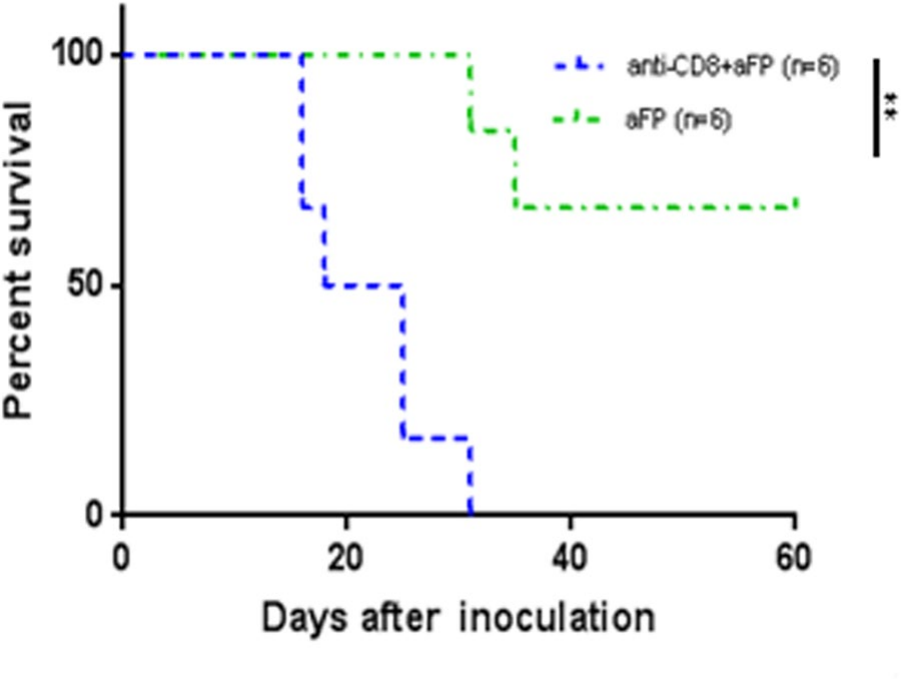
Tumor Survival Curves after aFP Treatment with Anti-CD8 Depletion Antibody. To investigate whether adaptive immunity is necessary to eradicate cancer cells after aFP, tumor inoculation was performed with anti-CD8 depletion antibody to ablate CD8+ T cells in the mouse in the one tumor model. Anti-CD8 depletion antibodies were administered intraperitoneally at a dose of 200 µg per mouse every 3 days from one day before tumor inoculation to removal of mice as endpoint. The graph shows Kaplan-Meier survival curves of mice receiving tumor inoculation and anti-CD8 depletion antibody. The significance values for the difference between the survival curves are: anti-CD8+ aFP vs. aFP: *P* < 0.005.

To investigate whether adaptive immunity is necessary to eradicate cancer cells after aFP, we repeated the experiment with anti-CD8 depletion antibody to ablate CD8+ T cells in the mouse. As shown in Fig. 3, treatment of aFP in absence of CD8+ T cells failed to prevent tumor growth and eventual euthanasia, while 4 out of 6 mice in control group, with no CD8 depletion, survived. (P < 0.005; Fig. 3). This observation indicates that adaptive immunity is necessary to eradicate cancer cells after aFP.

### aFP + anti-PD-1 inhibitor combination therapy induces development of systemic anti-tumor immunity

**Figure 4:**
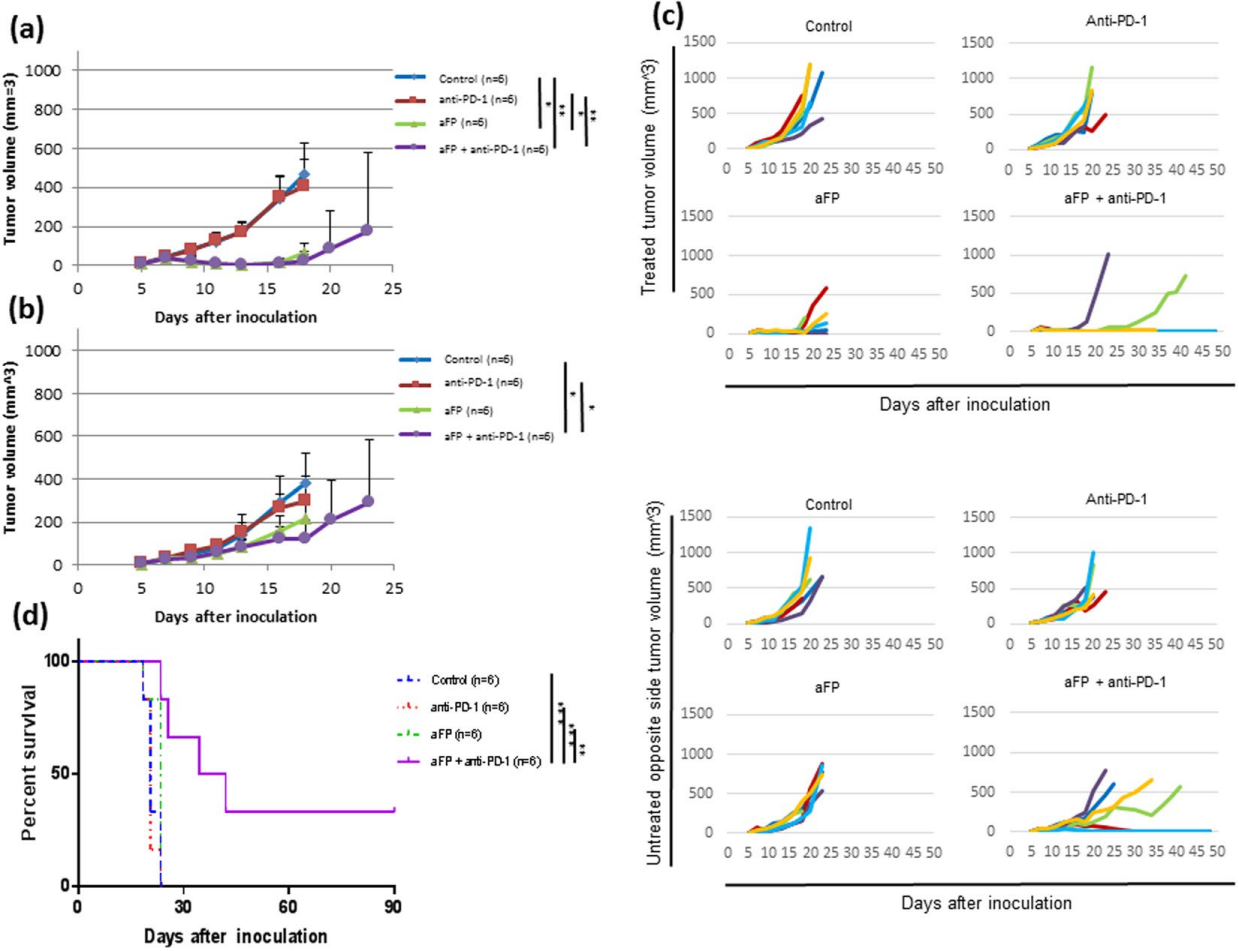
Tumor Volume and Survival Curves after Treatment in the Two Tumor Mouse Model. To investigate whether aFP treatment could induce systemic anti-tumor immunity, we established a mouse model which has one tumor on each hind leg with the aFP-treated tumor on the left leg and observed the growth of both tumors. aFP laser irradiation was performed 7 days after tumor inoculation. Anti-PD-1 blocking antibodies were administered intraperitoneally at a dose of 200 µg per mouse on days 7, 9, 11, 13, and 15 after tumor cell inoculation. (**a**) Average of tumor volume curves on the treated legs of mice in the control, anti-PD-1, aFP and aFP + anti-PD-1 groups after tumor inoculation. (**b**) Average of tumor volume curves on the untreated contralateral legs of mice in the control, anti-PD-1, aFP and aFP + anti-PD-1 groups after tumor inoculation. (**c**) Individual tumor volume curves in the control, anti-PD-1, aFP and aFP + anti-PD-1 groups after tumor inoculation. (**d**) Kaplan-Meier survival curves of mice receiving tumor inoculation. The significance values for the difference between the survival curves are: Control vs. aFP + anti-PD1: *P* < 0.005, Anti-PD-1 vs. aFP + anti-PD1: *P* < 0.005, aFP vs. aFP + anti-PD1: *P* < 0.01. The bars represent SD. *P < 0.05, **P < 0.01, ***P < 0.005.

To investigate whether aFP treatment could induce systemic anti-tumor immunity, we established a two tumor mouse model, which has a tumor on both hind legs, with the aFP-treated tumor on the left leg and observed the growth of both tumors. aFP laser irradiation was performed 7 days after tumor inoculation. The aFP significantly led to a reduction in tumor volume and growth rate of aFP-treated tumors after the treatment (Fig. 4a and c). Regarding the untreated contralateral tumors, we found that the aFP + anti-PD-1 combination therapy led to a significant reduction in growth rate after the treatment (Fig. 4b and c). Moreover the untreated contralateral tumors shrank in 2 of the 6 mice in the aFP + anti-PD-1 group completely, but such shrinkage did not occur in the rest of the groups (Fig. 4c and d). The significance value for the difference between the survival curves are: control vs. aFP + anti-PD-1 (*p* < 0.005), anti-PD-1 vs. aFP + anti-PD-1 (*p* < 0.005), aFP vs. aFP + anti-PD-1 (*p* < 0.01).

### aFP induces recruitment of CD3+, CD4+, CD8+ and epitope specific CD8+ T lymphocytes but increases infiltration of Treg into the untreated contralateral tumors in the two tumor mouse model

**Figure 5:**
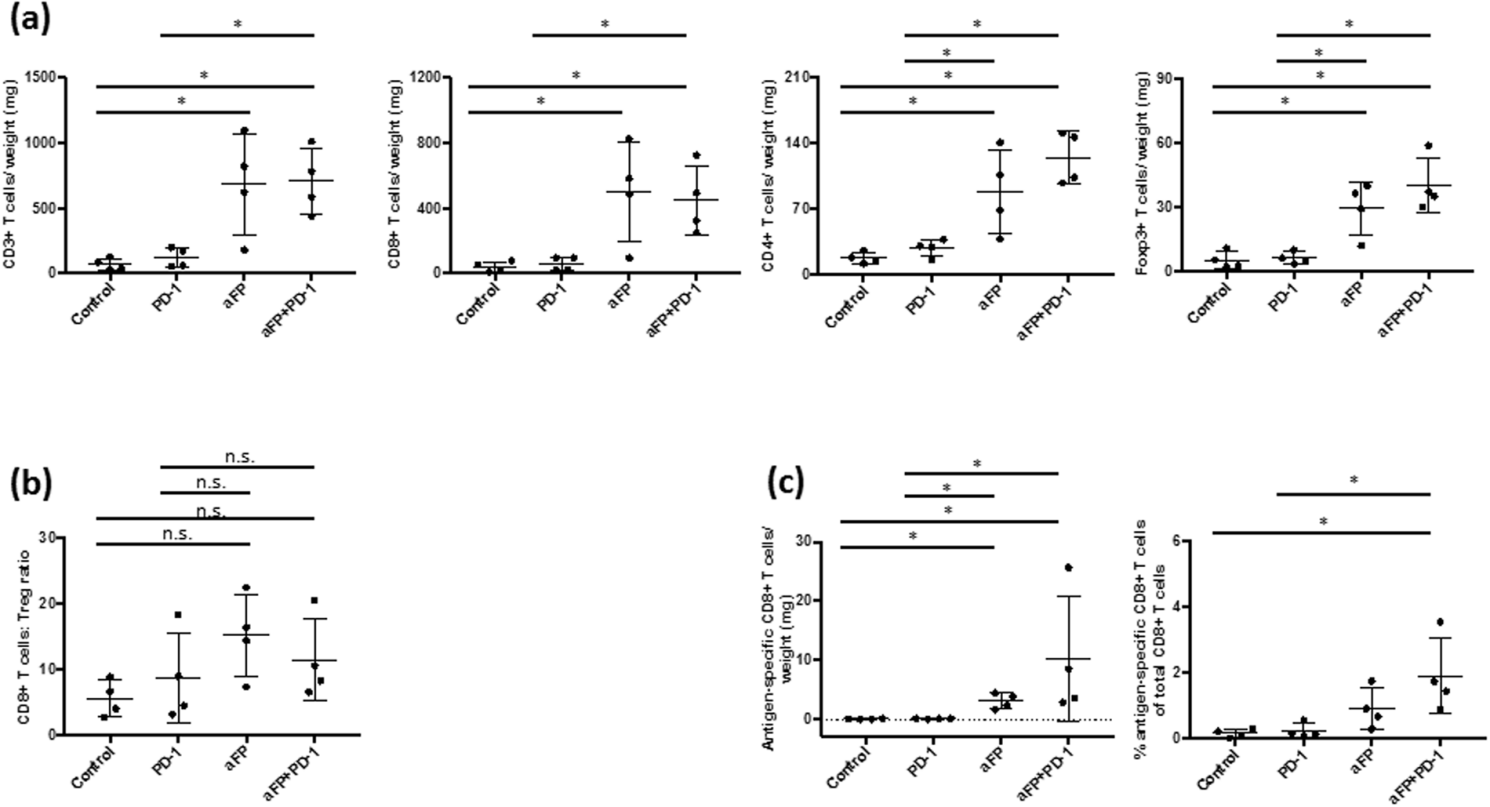
Flow Cytometric Analysis for Tumor Infiltrating Lymphocytes 12 Days after aFP Treatment in the Two Tumor Mouse Model. Flow cytometry analysis was performed 12 days after aFP treatment to investigate the number of CD3+, CD4+, and CD8+, epitope specific CD8+ T cells and Tregs expressing CD4, and Foxp3 inside the untreated contralateral tumor when the untreated contralateral tumor in the aFP + anti-PD-1 group shrank. (**a**) Proportion of CD3+, CD8+ and CD4+ T cells and Treg normalized to tumor weight. (**b**) Ratio of CD8+ T cells to Tregs (CD4+ Foxp3+). (**c**) Proportion of antigen specific CD8+ T cells normalized to tumor weight and the percentage of antigen specific CD8+ T cells of total CD8+ T cells. The bars represent SD. *P < 0.05.

To investigate why the untreated contralateral tumor in the aFP group did not shrink even though epitope specific CD8+ T lymphocytes were developed in the aFP-treated tumor, we measured the number of CD3+, CD4+, CD8+, epitope specific CD8+ T cells, and Foxp3+ Tregs inside the untreated contralateral tumor using flow cytometry 12 days after aFP treatment. We chose to measure on 12 days after aFP treatment because on this day the untreated contralateral tumor in the aFP + anti-PD-1 group shrunk significantly (aFP-treated tumors in the two groups shrank within 5 days after aFP treatment). We found that CD4+, CD8+ and epitope specific CD8+ T cells increased per tumor weight in the aFP-treated groups (CD3/weight and CD8/weight: control vs. aFP, control vs. aFP + anti-PD-1 and anti-PD-1 vs. aFP + anti-PD-1 (*p* < 0.05); CD4/weight and epitope specific CD8+ T cells: control vs. aFP, control vs. aFP + anti-PD-1, anti-PD-1 vs. aFP and anti-PD-1 vs. aFP + anti-PD-1 (*p* < 0.05; Fig. 5a and c). Moreover the percentage of AH1 specific CD8+ T cells compared to total CD8+ T cells increased in the aFP-treated groups (control vs. aFP and control vs. aFP + anti-PD-1 (*p* < 0.05; Fig. 5c). However, we found that Tregs increased in the aFP-treated groups per tumor weight though there was no significant difference regarding Treg numbers in aFP-treated tumors in the all groups (Fig. 5a). Furthermore, there is no significant difference in the ratio of CD8+ T cells to Treg in the all groups (Fig. 5b).

Additionally to investigate the up-regulation of PD-L1 on tumor cells, which is mediating immune suppression to escape immune surveillance, we investigated the PD-L1 expression on the tumor cell using flow cytometry 5 days and 12 days after aFP treatment. We found that there were high percentage of PD-L1 positive cells in the tumor in the all groups 12 days after aFP treatment even though lower percentage of CT26WT cells were PD-L1 positive before inoculation (Supplementary Figure 1a). The percentage of PD-L1 positive cells increased as the tumor grew (Supplementary Figure 1b). These data suggest that aFP induces infiltration of epitope specific CD8+ T cells into the untreated contralateral tumor; however, the function of the cells was impaired by high percentage of PD-L1 positive cells, which increased as tumors grew. Therefore, unlike the aFP-treated tumor, the untreated contralateral tumor in the aFP group did not shrink.

### Antigen specificity is necessary for systemic anti-tumor immunity

**Figure 6:**
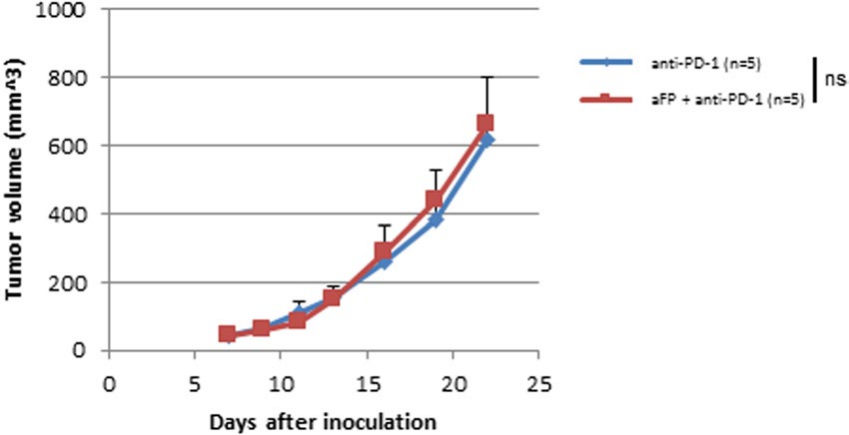
Tumor Volume Curves after Treatment in the Two Tumor Mouse Model with Mismatched Tumors, CT26WT and 4T1. To investigate whether the shrinkage of the untreated contralateral tumor in the aFP + anti-PD-1 group was due to antigen specific immunity induced by aFP or not, we performed experiments with two groups of mice that each had two mismatched tumors, CT26WT in left leg and 4T1 (murine mammary carcinoma) in right leg. The aFP group was treated with only anti-PD-1 inhibitor and the aFP + anti-PD-1 group was treated with aFP treatment in left leg (CT26WT) and anti-PD-1 inhibitor. The graph shows tumor (4T1) volume curves on untreated right leg of mice in the anti-PD-1 and aFP + anti-PD-1 groups after tumor inoculation. The bars represent SD. ns, not significant.

To investigate whether the shrinkage of the untreated contralateral tumor in the aFP + anti-PD-1 group was due to antigen specific immunity induced by aFP or not, we performed experiments with two groups of mice that each had two mismatched tumors, CT26WT in left leg and 4T1 (murine mammary carcinoma) in right leg. One group was treated with only anti-PD-1 inhibitor and the other group was treated with aFP treatment in the left leg (CT26WT) and anti-PD-1 inhibitor. There were no effects on the size or growth rate of the contralateral untreated tumors and survival mice in either group (Fig. 6).

## Discussion

This study demonstrates significant tumor size reduction and initiation of a systemic, tumor specific immune response induced by FP laser irradiation against a poorly immunogenic tumor^13^. A large range of clinical indications currently make use of FP, yet the ability of FP to induce tumor regression is unexpected, because FP treatments are generally applied to achieve localized tissue effects within the treatment area such as in the stimulation of wound healing and tissue regeneration.

There are reported studies that utilize tumor associated antigen (TAA) properties of the tumor to show anti-tumor activity of cancer therapies^17^. However, the antigen expression on the cell in a normal state is not sufficient to stimulate an immune response to prevent tumor growth^9^. Therefore, in the present study, we employed aFP because we hypothesized it would produce thermal denaturation, potentially releasing TAA and enhancing anti-tumor immunity in order for the cytotoxic T lymphocytes to recognize and bind the immunodominant peptide epitope derived from the antigens on MHC class I as it simultaneously creates MTZ with intact interspersed tissue. The total MTZ/tumor volume ratio was adjusted to approximately 5% of inoculated tumors, thereby aiming to enhance inflammation while avoiding the direct killing of the tumor bulk. Mroz *et al*. successfully reported laser irradiation with PDT to treat mouse subcutaenous immunogenic CT26.CL25 colon carcinoma cells, which was artificially transduced with lacZ gene to express an immunodominant antigen (beta-gal) induced antigen-specific immune response^9^. Moreover, PDT induces the immune response even in poorly immunogenic CT26WT colon carcinoma cells, which are beta-gal negative parental carcinoma of CT26.CL25 cells^10–12^. Therefore, we were motivated to investigate whether aFP could induce anti-tumor immunity against clinically relevant mouse tumor models and found that aFP treatment against the poorly immunogenic tumors could induce anti-tumor immunity and produce improvement in long-term survival. The CT26WT colon carcinoma cells we employed in this study have TAA in a single peptide known as AH-1, a non-mutated nonamer derived from the envelope protein (gp70) of an endogenous ecotropic murine leukemia provirus^18^. Mroz *et al*. showed that PDT induced local remission of CT26.CL25 tumors as well as a systemic tumor-specific immune response derived from release of TAA (beta-gal) from the tumor by PDT inducing epitope specific CD8+ T cells^9^. In our present study the pentamer staining revealed increase of TAA (AH1) specific CD8+ T cells per tumor weight in the aFP-treated groups. The result suggests aFP can also release TAA and induce anti-tumor immunity. The effectiveness of aFP is associated with thermal protein denaturation^1^. Reports have shown that thermal denaturation induces gene expression^19,20^ and up-regulates chemokines^21^. Furthermore, it leads to release of damage-associated molecular patterns (DAMPs)^22^ and induces innate immunity^23,24^. These facts suggest thermal denaturation induced by aFP initiated a strong immune response locally, resulting in induction of epitope specific CD8+ T cell response.

Interestingly, even though epitope specific CD8+ T cells infiltrated into both sides of the tumors in the aFP group in the two tumor mouse model, there were no surviving mice in the group. We assume the reason for this is the abundant presence of PD-L1+ tumor cells that can block systemic CD8+ T cell immunity mounted by aFP treatment in the untreated contralateral tumor cells 12 days after aFP treatment since it is indicated by the survival benefit when using anti-PD-1 inhibitor in the aFP + anti-PD-1 group.

In addition to blocking the PD-1/PD-L1 axis, PD1 inhibitor boosts clonal expansion of antigen specific CD8+ T cells^25^. Therefore, there were tendencies that AH1 epitope specific CD8+ T cells compared to total CD8+ T cells increased more in the aFP + anti-PD-1 group than in the aFP group. Even though the PD-1/PD-L1 axis was blocked by anti-PD-1 inhibitor and the high frequency of the epitope specific CD8+ T cells was observed, complete remission of the untreated contralateral tumor was observed in fewer cases than the aFP-treated tumors in the aFP + anti-PD-1 group in the two tumor mouse model. We assume the reason is the ratio values of CD8+ T cells compared to Treg in the untreated contralateral tumors are lower than in the aFP-treated tumors.

In conclusion, we used aFP to induce anti-tumor immunity. Despite the small degree of thermal injury to the tumor, aFP induced a long-lasting anti-tumor immunity. Moreover, combining aFP therapy with an anti-PD-1 inhibitor further boosts the systemic immunity. These effects might be mediated by an increase in the number of tumor antigen-specific cytotoxic T cells, induced by aFP treatment. Thus, such a therapeutic strategy might achieve significant efficacy in poorly immunogenic cancers of colon, breast, lung and other organs.

## Materials and Methods

### Cell lines

CT26WT murine colon cancer cell lines (ATCC, Mannassas, VA) were cultured in RPMI Medium supplemented with 10% heat-inactivated fetal bovine serum, penicillin (100 U/mL) and streptomycin (100 mg/mL) (all from Sigma-Aldrich, Natick, MA) at 37 °C in 5% CO_2_. Culturing was performed in 75 cm^2^ flasks (Falcon, Invitrogen, Carlsbad, CA).

### Animals

Six-week-old female BALB/c mice (Charles River Laboratories, Boston, MA) were used for the study. The care and handling of the animals was done in accordance with a protocol approved by the Subcommittee on Research Animal Care (IACUC) at Massachusetts General Hospital (MGH).

### Animal tumor model

**Figure 7:**
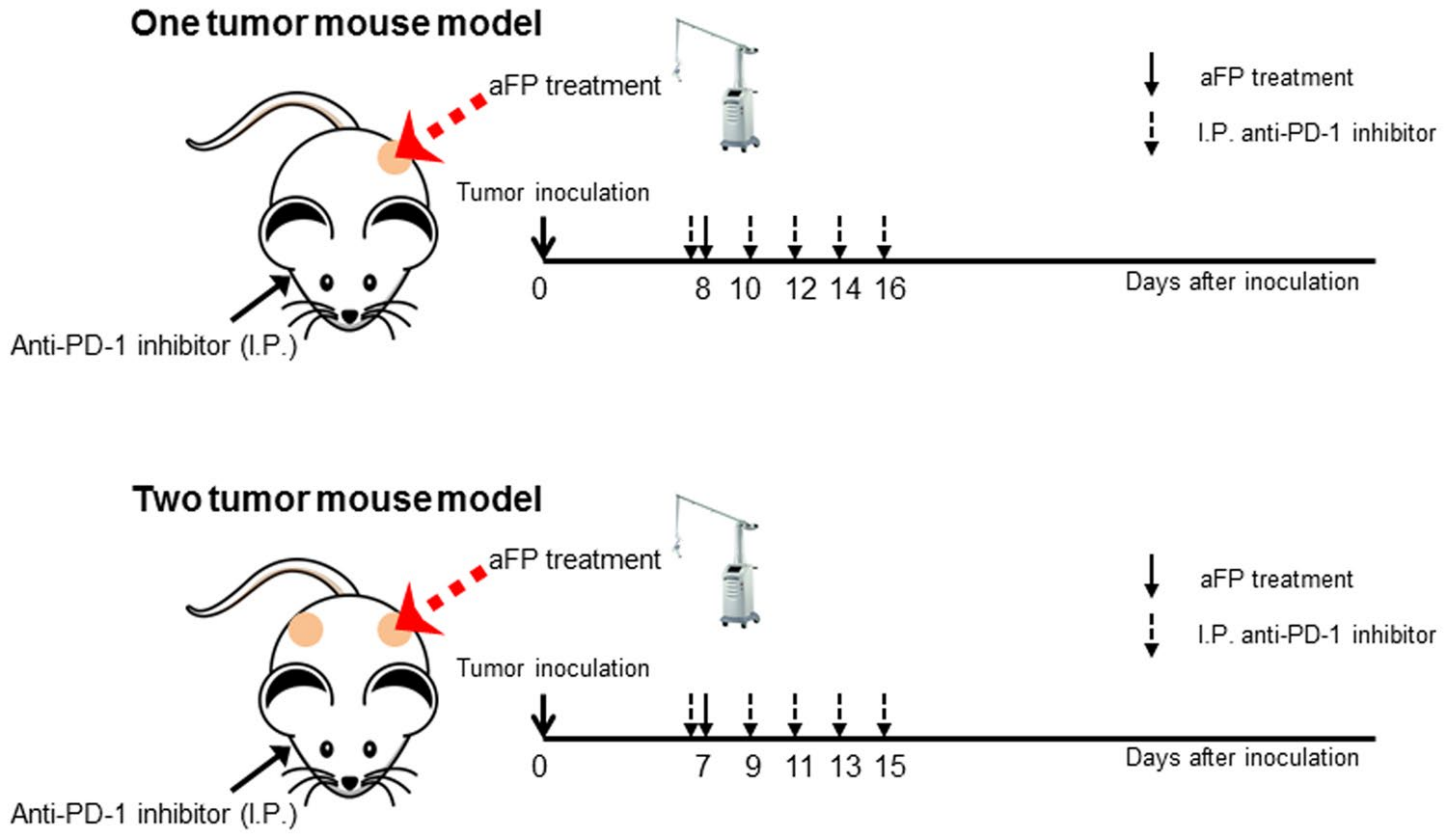
Experimental Design Scheme of *In Vivo* aFP, Anti-PD-1 Inhibitor.

Mice were inoculated unilaterally at the left leg or bilaterally with 3.5 × 10^5^ CT26WT subcutaneously into the depilated thigh, after being anesthetized through intraperitoneal injection of a cocktail of ketamine (90 mg/kg) and xylazine (10 mg/kg). Anti-PD-1 blocking antibodies (29 F.1A12; BioXCell,West Lebanon, NH) were administered intraperitoneally at a dose of 200 µg per mouse on days 8, 10, 12, 14, and 16 in the one tumor mouse model, and days 7, 9, 11, 13, and 15 in the two tumor mouse model after tumor cell inoculation (Fig. 7). Anti-CD8 depletion antibodies (2.43; BioXCell,West Lebanon, NH) were administered intraperitoneally at a dose of 200 µg per mouse every 3 days from one day before tumor inoculation to removal of mice as endpoint. The tumor volume was determined 3 times per week by measuring the longest dimension and orthogonal dimension of the tumor with vernier calipers. Tumor volumes were calculated according to the formula volume = 4π/3 × [(a + b)/4]^3^, where a and b represent the long and short axis lengths, respectively. If the tumor volume exceeded 700 mm^3^ or showed severe ulceration, the mouse was removed from the study as endpoint.

### Fractional CO_2_ laser irradiation

Ablative fractional laser exposure was performed on the tumor site at day 7 or 8 after tumor cell inoculation (Fig. 7). At that time, the tumors have reached a typical diameter of about 5–6 mm. Exposures were performed with an Ultrapulse Encore CO_2_ laser (Lumenis Inc, Yokneam, Israel). A single aFP treatment was performed within a treatment area of 6 × 6 mm, with a pulse energy of 100 mJ, at a nominal density of 5% (i.e., 5% within the treatment area surface was irradiated), and a pulse repetition frequency of 300 Hz. No skin cooling was applied and the anesthesia was performed by intraperitoneal injection of a cocktail of ketamine (90 mg/kg) and xylazine (10 mg/kg).

### Rechallenge

Mice surviving 90 days after tumor inoculation were rechallenged with matched tumor cells (3.5 × 10^5^ CT26WT) subcutaneously at the contralateral (right) leg from previously aFP-treated side. Age-matched naive mice were inoculated with the same number of the cells in the right leg as the controls. The inoculated mice were monitored for another 60 days to confirm tumorigenesis.

### Flow cytometry analysis

To confirm whether CD3+ and CD8+ lymphocyte, and regulatory T cell (Treg) numbers were affected by aFP, and the PD-L1 expressed on the tumor cells, flow cytometry analysis was performed. For leukocyte isolation from tumor, fresh CT26WT tumors were dissociated mechanically filtering through 70 μm strainer on 6 well culture plate with DNaseI (10 μg/ml, Roche; Nutley, NJ) and collagenase (10 mg/ml, Life technologies) and incubated 60 minutes at 37 °C. The dissociated cells were positively and negatively selected for CD45 surface expression using CD45 MicroBeads and magnetic-activated cell sorting (MACS) system (Miltenyi Biotec Inc., Auburn, CA). Then the positively selected cells were stained with antibodies CD3-FITC (145-2C11), CD4-APC-eFluor780 (RM4-5), CD8a-PE/CY7 (53-6.7) for 30 minutes at 4 °C after they were blocked at 4 °C for 15 minutes using CD16/CD32 antibody (93) and negatively selected cells were stained PD-L1-PerCPeFluor 710 (MIH5) and CD45-PE (30-F11; all from eBioscience; Santa Clare, CA) for 30 minutes at 4 °C. Following staining for surface markers, the negatively selected cells were fixed in 2% paraformaldehyde at room temperature for 20 minutes and the positively selected cells were fixed and permeabilized by Foxp3/ Transcription Factor Staining Buffer Set (eBioscience) according to the manufacturer’s instructions at room temperature for 30 minutes. After the permeabilization the cells were stained with Foxp3-APC antibody (FJK-16s; eBioscience; Santa Clare, CA) at 4 °C over night. The next day the stained cells were analyzed on FACSCanto (BD Bioscience, Franklin Lakes, NJ).

### Pentamer staining

To confirm tumor associate antigen (TAA) of CT26WT specific CD8+ lymphocytes were elicited by aFP treatment, we stained isolated leukocytes from tumor with AH1 epitope (SPSYVYHQF) specific pentamer (ProImmune, Oxford, UK) co-staining with anti CD8a-PE/CY7 (53-6.7), CD4-APC-eFluor780 (RM4-5), CD3-FITC (145-2C11) and Foxp3-APC antibodies (FJK-16s; all from eBioscience; Santa Clare, CA). The assay was conducted following manufacturer’s instructions.

### Statistics

All experiments were repeated at least once. All statics analyses were performed with GraphPad Prism 7.0 (GraphPad Software). All values are expressed as the mean ± SD. Flow cytometric results were compared with a two-tailed Mann-Whitney test. Tumor growth curves were compared with a two-tailed Wilcoxon rank-sum test. Survival analysis was performed using the Kaplan-Meier method and a log-rank test. Values of P < 0.05 were considered statistically significant.

## Electronic supplementary material

Supplemental figure

## References

[1] Manstein D, Herron GS, Sink RK, Tanner H, Anderson RR (2004). Fractional photothermolysis: a new concept for cutaneous remodeling using microscopic patterns of thermal injury. Lasers Surg Med.

[2] Khan MH, Sink RK, Manstein D, Eimerl D, Anderson RR (2005). Intradermally focused infrared laser pulses: thermal effects at defined tissue depths. Lasers Surg Med.

[3] Hantash BM, Bedi VP, Chan KF, Zachary CB (2007). *Ex vivo* histological characterization of a novel ablative fractional resurfacing device. Lasers Surg Med.

[4] Tierney EP, Kouba DJ, Hanke CW (2009). Review of fractional photothermolysis: treatment indications and efficacy. Dermatol Surg.

[5] Tierney EP, Hanke CW, Petersen J (2012). Ablative fractionated CO2 laser treatment of photoaging: a clinical and histologic study. Dermatol Surg.

[6] Prignano F (2009). Fractional CO2 laser: a novel therapeutic device upon photobiomodulation of tissue remodeling and cytokine pathway of tissue repair. Dermatol Ther.

[7] Kim HW, Chang SE, Kim JE, Ko JY, Ro YS (2014). The safe delivery of fractional ablative carbon dioxide laser treatment for acne scars in Asian patients receiving oral isotretinoin. Dermatol Surg.

[8] Trelles MA, Velez M, Gold MH (2010). The treatment of melasma with topical creams alone, CO2 fractional ablative resurfacing alone, or a combination of the two: a comparative study. J Drugs Dermatol.

[9] Mroz P, Szokalska A, Wu MX, Hamblin MR (2010). Photodynamic therapy of tumors can lead to development of systemic antigen-specific immune response. PLoS One.

[10] Rocha LB, Gomes-da-Silva LC, Dabrowski JM, Arnaut LG (2015). Elimination of primary tumours and control of metastasis with rationally designed bacteriochlorin photodynamic therapy regimens. Eur J Cancer.

[11] Wachowska M (2014). 5-Aza-2′-deoxycytidine potentiates antitumour immune response induced by photodynamic therapy. Eur J Cancer.

[12] Yeung HY, Lo PC, Ng DK, Fong WP (2017). Anti-tumor immunity of BAM-SiPc-mediated vascular photodynamic therapy in a BALB/c mouse model. Cell Mol Immunol.

[13] Griswold DP, Corbett TH (1975). A colon tumor model for anticancer agent evaluation. Cancer.

[14] Pardoll DM (2012). The blockade of immune checkpoints in cancer immunotherapy. Nature reviews. Cancer.

[15] Sharma P, Allison JP (2015). The future of immune checkpoint therapy. Science.

[16] Iwai Y, Terawaki S, Honjo T (2005). PD-1 blockade inhibits hematogenous spread of poorly immunogenic tumor cells by enhanced recruitment of effector T cells. International immunology.

[17] Mroz P, Vatansever F, Muchowicz A, Hamblin MR (2013). Photodynamic therapy of murine mastocytoma induces specific immune responses against the cancer/testis antigen P1A. Cancer Res.

[18] Huang AY (1996). The immunodominant major histocompatibility complex class I-restricted antigen of a murine colon tumor derives from an endogenous retroviral gene product. Proceedings of the National Academy of Sciences of the United States of America.

[19] Sonna LA (2004). Exertional heat injury and gene expression changes: a DNA microarray analysis study. Journal of applied physiology.

[20] Sonna LA, Fujita J, Gaffin SL, Lilly CM (2002). Invited review: Effects of heat and cold stress on mammalian gene expression. Journal of applied physiology.

[21] Mackanos MA, Helms M, Kalish F, Contag CH (2011). Image-guided genomic analysis of tissue response to laser-induced thermal stress. Journal of biomedical optics.

[22] Hazeldine J, Hampson P, Lord JM (2016). The diagnostic and prognostic value of systems biology research in major traumatic and thermal injury: a review. Burns & trauma.

[23] Krysko DV (2012). Immunogenic cell death and DAMPs in cancer therapy. Nature reviews. Cancer.

[24] Zelenay S, Reis e Sousa C (2013). Adaptive immunity after cell death. Trends Immunol.

[25] Tumeh PC (2014). PD-1 blockade induces responses by inhibiting adaptive immune resistance. Nature.

