# Supplementary material for: Fractional Laser Releases Tumor-Associated Antigens in Poorly Immunogenic Tumor and Induces Systemic Immunity
Source: Sci Rep. 2017 Oct 6;7:12751. doi: 10.1038/s41598-017-13095-8 (PMC5630620; doi:10.1038/s41598-017-13095-8)
Supplement: Supplementary file 1 — Supplemental figure [file 41598_2017_13095_MOESM1_ESM.pdf]

Fractional Laser Releases Tumor-Associated Antigens in Poorly Immunogenic Tumor and Induces Systemic Immunity

Masayoshi Kawakubo[1,2 *], Trevor J. Cunningham[1,2], Shadmehr Demehri[1,2], Dieter Manstein[1,2]

[1]Cutaneous Biology Research Center, Department of Dermatology, Massachusetts General Hospital Research Institute, Charlestown, MA 02129, USA

[2]Harvard Medical School, Boston, MA

[*] Correspondence: mkawakubo@mgh.harvard.edu

Conflict of interest statement: the authors have no conflict of interest.

Supplementary Information

Supplementary figure

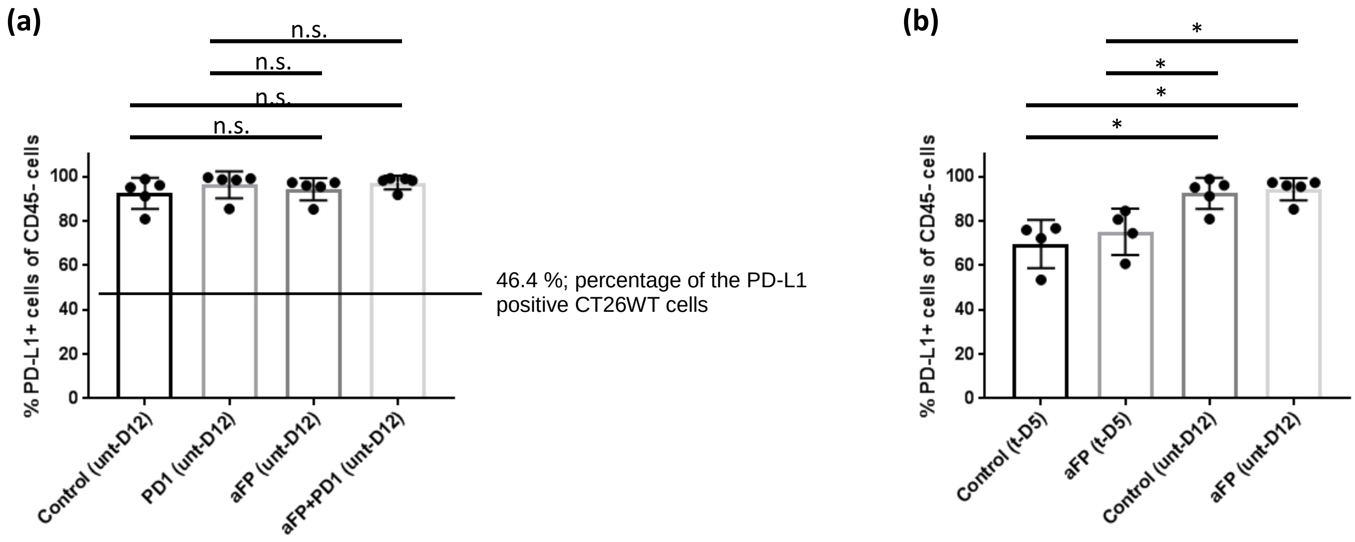

Supplementary figure 1. Flow Cytometric Analysis for PD-L1 Expression of the CT26WT Tumor in the Two Tumor Mouse Model.

To investigate the up-regulation of PD-L1 on tumor cells which is mediating immune suppression to escape immune surveillance, we measureed PD-L1 expression on CT26WT tumor cell using flow cytometry. (a) % PD-L1+ cells of total CD45- cells in the untreated contralateral tumor in the two tumor mouse model on 12 days after aFP treatment. (b) % PD-L1+ cells of total CD45- cells in control and aFP group in aFP-treated tumor in the one tumor mouse model 5 days after aFP treatment, and in the untreated contralateral tumor in the two tumor mouse model 12 days after aFP treatment. The bars represent SD. * $P < 0.05$
